# Horizontal semicircular canal benign paroxysmal positional vertigo treated by acupuncture and moxibustion: A case report

**DOI:** 10.1097/MD.0000000000036032

**Published:** 2023-11-24

**Authors:** Ying Cai, Qi Wen Zhang, Shan Li, Quan Ai Zhang

**Affiliations:** a The Third Clinical College, Zhejiang Chinese Medical University, Hangzhou, China; b Department of Acupuncture and Moxibustion, The Third Affiliated Hospital of Zhejiang Chinese Medical University, Hangzhou, China; c Research Center of Experimental Acupuncture Science, Tianjin University of Traditional Chinese Medicine, Tianjin, China

**Keywords:** acupuncture, horizontal semicircular canal benign paroxysmal positional vertigo, vertigo, wheat grain moxibustion

## Abstract

**Rationale:**

Horizontal semicircular canal benign paroxysmal positional vertigo (HSC-BPPV) is a second common canal of Benign Paroxysmal Positional Vertigo (BPPV); its actual incidence may have been underestimated because of its complex pathogenesis. Although the canalith repositioning maneuver is the treatment of choice, it has a high recurrence rate, affecting some patients’ lives and psychology. We submit a case report describing acupuncture and wheat grain moxibustion treatment for HSC-BPPV.

**Patient concerns:**

A 70-year-old patient with HSC-BPPV had low acceptability of the otolith repositioning treatment strategy and reported intolerance during the procedure. He turned to acupuncture as a result of recurrent attacks of vertigo.

**Diagnoses:**

Horizontal semicircular canal benign paroxysmal positional vertigo.

**Interventions:**

The intervention project was acupuncture followed by wheat grain moxibustion treatment, administered once every 2 days, 3 times a week. The whole treatment period lasted for 2 months.

**Outcomes:**

The patient’s clinical symptoms of vertigo improved significantly after 8 weeks of acupuncture and wheat grain moxibustion treatment. The Dizziness Handicap Inventory (DHI) and Visual Vertigo Analogue Scale (VVAS) scores decreased, thus verifying that the severity of vertigo was reduced.

**Lessons:**

This brief clinical report suggests that acupuncture therapy may be a complementary option for treating HSC-BPPV.

## 1. Introduction

Horizontal semicircular canal-benign paroxysmal positional vertigo (HSC-BPPV), also known as lateral semicircular canal benign paroxysmal positional vertigo (LSC-BPPV), accounts for approximately 10% to 30% of all patients with benign paroxysmal positional vertigo.^[1]^ Horizontal semicircular canal-benign paroxysmal positional vertigo (HSC-BPPV) has a long disease course, can easily be misdiagnosed, and has a high recurrence rate. This condition is also associated with complex pathophysiology and difficult clinical lateralization.^[2]^ The Gufoni maneuver, the Forced prolonged position (FPP), the Barbecue maneuver, and the Zuma maneuver are among the canalith repositioning maneuvers now the most effective evidence-based treatments for HSC-BPPV.^[3–5]^ Although therapeutically beneficial, manipulative resetting is inappropriate for all HSC-BPPV patients. For instance, the Gufoni maneuver is not recommended for patients with poor tolerance for vertigo and somatic disorders, as this technique can result in the recurrence of vertigo, persistent residual symptoms, and abnormal otolith function. The Barbecue maneuver, on the other hand, can be uncomfortable during the repositioning process.^[6–8]^ Long-term treatment, particularly psychotropic medicines such as benzodiazepines, can also increase the risk of falls and cognitive difficulties in patients with HSC-BPPV.^[1]^

In many previous clinical studies and trials, Chinese medicine has effectively treated vertigo.^[9–12]^ Based on this evidence, we hypothesized that acupuncture might effectively treat HSC-BPPV. Here, we report the case of a 70-year-old patient diagnosed with HSC-BPPV. He received acupuncture and wheat grain moxibustion to investigate the effectiveness of acupuncture for the treatment of HSC-BPPV.

## 2. Clinical report

A 70-year-old man presented to the clinic with recurrent vertigo attacks for 1 year with exacerbation for 1 month. The patient complained of recurrent episodes of vertigo in the head, often with changes in head position, accompanied by stuffiness in the ears, cold sweats, and nausea. The patient had undergone manual repositioning treatment in other hospitals but developed nausea, vomiting, and other intolerance symptoms during the repositioning process. He refused subsequent repositioning treatment. His symptoms were relieved after taking betahistine mesylate tablets (Merislon, Eisai Co., Ltd., China) at an oral dose of 6 mg twice a day. However, vertigo still recurred and affected his daily activities. The patient had a history of hypertension but no head trauma, cervical radiculopathy, cerebrovascular disease, or otology. The patient was normal on physical examination and in the gaze test. The Supine Roll test showed variable horizontal nystagmus, which lasted for about 22s in the same direction as her head movement to the right and left; in particular, the right-side nystagmus was stronger than the left-side. The Dix–Hallpike test showed no obvious nystagmus. The vestibulo-ocular reflex test suggested a deduction in the left horizontal semicircular gain. Before treatment, the patient scored 82.2 on the Visual Vertigo Analogue Scale (VVAS) and VVAS positive, which measures the severity of vertigo (the Scoring criteria are shown in Table 1), and 82 on the Dizziness Impairment Inventory (DHI). Based on the patient’s clinical presentation, examination, and evaluation criteria, he was definitively diagnosed with HSC-BPPV.

**Table 1:** Visual Vertigo Analogue Scale (VVAS) scoring criteria.

Please select a corresponding number to represent your dizziness degree, with zero (0) representing no dizziness and ten (10) representing extreme dizziness or activity avoided due to dizziness.
1. Walking in supermarket
2. Passenger in a car
3. Under fluorescent lights
4. At an intersection
5. Being in shopping centers
6. Going on escalators
7. At movies or the theater
8. On patterned floors
9. Watching television

The subjects were classified as VVAS positive if 2 or more items were rated above zero on the analogue scale. We also classified the VV severity; a score of 0 indicates that a patient did not experience VV, whereas a score of 90–100 would indicate severe visual vertigo.

**Figure 1.**
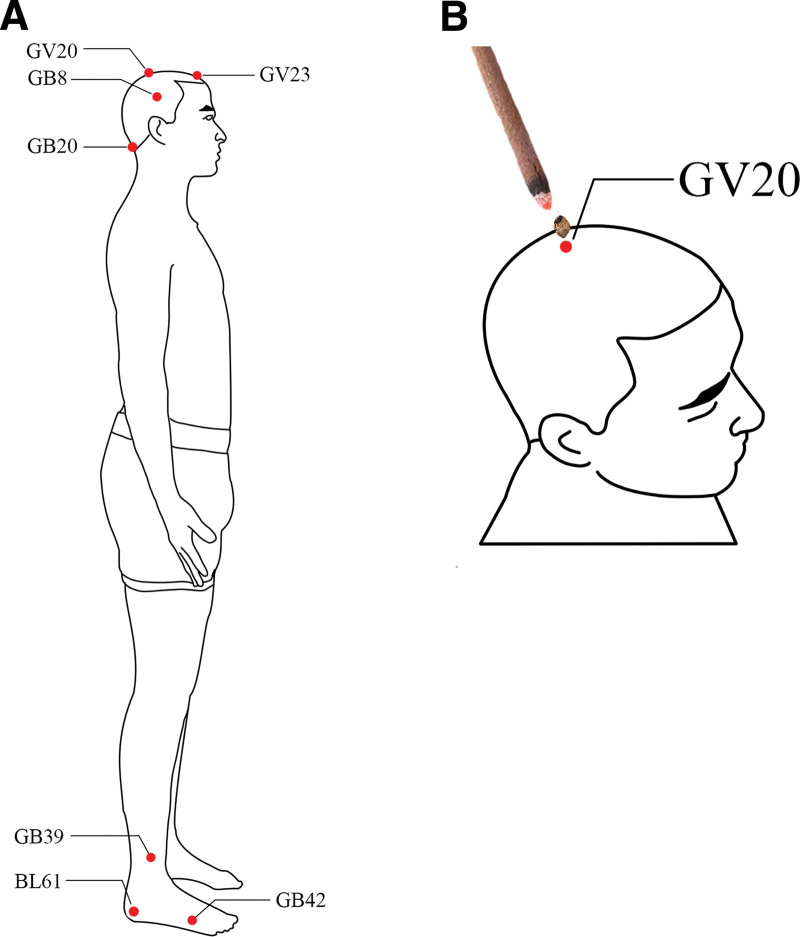
Diagrammatic presentation of acupuncture and moxibustion protocol. (A) Image showing red dots marked on the human body representing the locations of acupuncture points. (B) Image showing wheat-grain moxibustion at the GV20 acupoint. Wheat-grain moxibustion was ignited with a joss-stick to stimulate this acupoint.

The patient received acupuncture treatment for 2 months, 3 times a week. After routine needling of bilateral GB20 (FengChi), BL61 (PuCan), GB42 (DiWuHui), GV23 (ShangXing), and GB8 (ShuaiGu) acupoints, the needle was left in place for thirty minutes, and then withdrawn slowly. Subsequently, wheat grain moxibustion was performed on GV20 (BaiHui) and GB39 (XuanZhong) acupoints. The specific wheat grain moxibustion protocol was as follows: (1) the patient keeps a suitable position; (2) a moxa cone the size of a grain of wheat was put on the GV20 (BaiHui)and GB39 (XuanZhong) directly burned; (3) when the patient felt pain and removed immediately and repeated 5 times at each acupuncture point (see Fig. 1).

**Figure 2.**
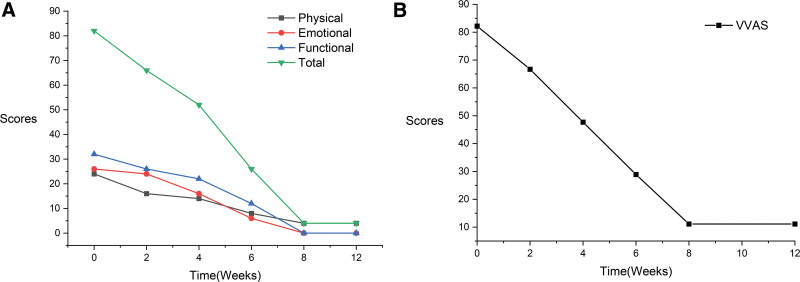
The Dizziness Handicap Inventory (DHI) score and the Visual Vertigo Analogue Scale (VVAS) score of patient before and after treatment. (A) Image showing that the patient’s emotional, functional, and physical scores of DHI were lower than before treatment. (B) Image showing that the VVAS scores decreased in this patient after treatment and during follow-up.

No adverse events occurred throughout the treatment period. After 2 weeks of acupuncture and wheat grain moxibustion treatment, the patient reported less dizziness when he turned his head up and down to the left. However, the dizziness persisted when lowering the head to the right. After 8 weeks of treatment, the DHI score improved by 78 points, the VV severity was 71, and the VVAS was positive (see Fig. 2A and B). The Supine Roll test revealed negative spontaneous nystagmus. The patient’s autonomic symptoms improved significantly (including nausea, vomiting, and cold sweats), balance capacity, and positive affect. At the telephone follow-up 3 months later, the patient reported that his vertigo symptoms had disappeared, and his daily living ability and quality of life were improved.

## 3. Discussion

In this case, a 70-year-old male patient diagnosed with HSC-BPPV, we chose acupuncture as a complementary treatment option because of the poor effect of otolith repositioning treatment and poor acceptance due to intolerance, including increased nausea and vomiting.

In this case, the acupuncture treatment protocol first called for a standard procedure before choosing which acupuncture points would receive wheat grain moxibustion. According to acupuncture theory, the bile meridian follows the ear and can treat ear diseases; therefore, the bile meridian acupoints GB20 (FengChi), GB42 (DiWuHui), and GB8 (ShuaiGu) were selected. The GB20 (FengChi) point is deep in the vertebral artery; acupuncture at this position can relieve vascular spasms, regulate blood circulation in the vertebrobasilar system, facilitate the formation of collateral circulation, and enhance blood flow to the inner ear.^[13]^ The greater occipital and auriculotemporal nerve branches are distributed under the area where the GB8 (ShuaiGu) acupoint is located. The GB8 (ShuaiGu) acupoint is suitable for ipsilateral dizziness, inner ear vertigo and tinnitus, and the treatment of cortical hearing impairment.^[14]^ Wheat grain moxibustion is a traditional Chinese direct moxibustion treatment method frequently used clinically in conjunction with acupuncture to treat diseases. In this case, wheat grain moxibustion on the GB39 (XuanZhong) and GV20 (BaiHui) acupoints improved lymphatic circulation in the inner ear and increased oxygen supply to the vascular endings of the inner ear.^[15]^ The superficial layer of the GV20 acupoint is rich in blood vessels and nerves. This acupoint’s deeper layer connects to the paracentral lobule and cerebral cortex; thus, acupuncture can directly stimulate nerves and vascular networks, increase blood flow to cerebral tissues, enhance blood oxygen saturation, and improve the state of cerebral hypoxia and ischemia. Acupuncture can also accelerate the absorption of lymphatic fluid in the ear.^[16]^ HSC-BPPV is prevalent in the elderly. In this case, the patient was a 70-year-old man, and we chose each acupoint to undergo wheat grain moxibustion 5 times to achieve appropriate stimulation. The results showed that acupuncture combined with wheat grain moxibustion achieved significant results in this case. Furthermore, the patient’s DHI and VVAS scores improved, and his mood changed to being more positive than previously.

The precise mechanism underlying the effect of acupuncture therapy on HSC-BPPV has yet to be fully validated, although several possible mechanisms have been considered. Firstly, acupuncture therapy improves vertigo symptoms by stimulating nerves in local tissues, by promoting the release of neuropeptides, and by enhancing local vasodilation and blood circulation.^[9]^ Secondly, acupuncture alleviates vertigo by increasing cerebral blood flow and reducing neurological dysfunction.^[17,18]^ Thirdly, acupuncture improves vertigo and vestibular nerve function by stimulating local acupuncture points, thus improving lymphatic circulation and end-ear oxygenation.^[19]^ Fourthly, long-term acupuncture can benignly regulate vestibular compensation and equalize the intensity of bilateral vestibular afferent signals, thus improving vertigo.^[20]^ Finally, the blood supply to the inner ear comes from the vagus artery, and almost 80% comes from the anterior inferior cerebellar artery. Cerebellar artery ischemia inevitably leads to inner ear ischemia and vertigo, and acupuncture accelerates inner ear blood circulation.^[21]^ The warm stimulation and light radiation effect of wheat grain moxibustion can dilate the membranous semicircular canal, accelerate hemolymph circulation, promote the movement of otoliths, and make the larger otoliths in the semicircular canal decompose into smaller otoliths; this facilitates self-dissipation, thus relieving the symptoms. Some studies show that thermal stimulation has anti-vertigo effects.^[22]^ Wheat moxibustion also reduces vertigo by enhancing blood circulation in the brain and improving ischemia in the brainstem’s reticular and vestibular nucleus area.^[23]^ The present case reports the treatment of HSC-BPPV with acupuncture combined with wheat grain moxibustion. This innovative study provides a basis for the effectiveness of acupuncture in the treatment of HSC-BPPV and also provides a complementary therapy for HSC-BPPV.

This case report has certain limitations. Since there are few studies on treating HSC-BPPV by acupuncture combined with moxibustion, there may be a lack of comprehensive understanding of the treatment mechanism and clinical efficacy in this case. At the same time, we only report one case, further studies with sufficient sample sizes are needed to provide evidence-based proof and explore the underlying mechanisms of action of this treatment.

## 4. Conclusion

The most effective treatment for HSC-BPPV is the canalith repositioning maneuver, but this procedure has inherent limitations. In this case, an older man who was intolerant to the canalith repositioning maneuver received acupuncture and moxibustion. The treatment result was positive, thus indicating that acupuncture and moxibustion may be helpful complementary therapy.

## Author contributions

**Conceptualization:** Ying-Ying Cai.

**Data curation:** Qi-wen Zhang, Shan-shan Li.

**Formal analysis:** Qi-wen Zhang, Shan-shan Li.

**Supervision:** Quan-ai Zhang.

**Writing – original draft:** Ying-Ying Cai.

**Writing – review & editing:** Quan-ai Zhang.
